# The Transmission of Tuberculosis through Meat and Milk

**Published:** 1901-12

**Authors:** 


### The Transmission of Tuberculosis Through Meat and Milk.

The above is the title of a well prepared paper by John J. Repp, V. M. D., of Iowa State College, concluded in the November 2nd number of *American Medicine*. After reviewing at length most of the literature on the subject, and incidentally taking Dr. Robert Koch severely to task, Dr. Repp ends his article as follows:

"The evidence presented here is:

1. That tuberculosis may be transmitted to animals through their eating the meat of certain other animals which are tuberculous, or by their being inoculated with it.

2. That tuberculosis may be transmitted to animals through their ingestion of the milk of certain cows which are tuberculous, or by their being inoculated with it, both when the udder of the cow is diseased and when it is healthy.

3. That, therefore, the meat and milk of certain tuberculous animals contain living, virulent tubercle bacilli.

4. That the tubercle bacilli of cattle are pathogenic for man.

5. That, therefore, the meat and milk of certain tuberculous animals are capable of producing tuberculosis in human beings who use these products as food.

### PRACTICAL CONCLUSIONS.

1. *In Regard to Meat.*—The meat of all food animals, especially cattle, is unfit for food when the animal is highly tuberculous; but is safe for food when the animal is only slightly or moderately tuberculous, especially so if the meat is well cooked, provided the tubercular tissues are eliminated.

2. *In Regard to Milk.*

(a) The milk of a cow with tuberculous udder is always dangerous for food unless it is well sterilized.

(b) The milk of tuberculous cows with healthy udders is sometimes dangerous for food unless well sterilized. We cannot tell except by experiment which is impracticable as a routine matter, when such milk is dangerous and when it is not. Hence, the milk of tuberculous cows without disease of the udder should always be looked upon with suspicion, and either not be used or be used only after sterilization.

(c) Tuberculous cows may be kept for breeding purposes, provided they are isolated, even from their own offspring, and their products sterilized before use; or,

(d) They may be slaughtered for food under conditions imposed by the conclusions stated above in regard to meat.

GENERAL CONCLUSION.

All legislation and regulation should favor the disposition of tuberculous animals as suggested above so far as meat and milk are concerned." R.

